# Genomic and Physiological Signatures of Evolution in ANAMMOX Bacteria

**DOI:** 10.1111/1758-2229.70197

**Published:** 2025-09-18

**Authors:** Roman G. Bielski, M. Ahsanul Islam

**Affiliations:** ^1^ Department of Chemical Engineering Loughborough University Loughborough UK

**Keywords:** activity, ANAMMOX bacteria, bioremediation, evolution, genomics

## Abstract

ANAMMOX bacteria are a group of strictly anaerobic bacteria that offer a sustainable and energy‐saving alternative to the conventional, energy‐intensive aerobic bacteria‐based fixed nitrogen removal technology from wastewater treatment plants. Since their discovery, research on ANAMMOX bacteria has expanded significantly, as reflected in the growing number of publications and patents. This review examines the evolutionary and physiological adaptations that have shaped these microorganisms, drawing from genomic and physiological studies. The distinct cellular architecture and membrane composition of ANAMMOX bacteria are discussed, alongside experimental literature that assesses their activity across various temperatures and pH conditions. Genomic analyses reveal significant differences between halophilic and non‐halophilic ANAMMOX bacteria, with halophiles sharing a unique set of genes absent in other species. Analysis of proteomic investigations further demonstrates evolutionary divergence, with halophilic strains exhibiting a bias toward acidic amino acids, as shown through principal component analysis. Together, these insights provide a comprehensive view of ANAMMOX bacterial evolution, linking genomic diversity to physiological adaptation.

## Graphical abstract

The cell layout of ANAMMOX bacteria is conserved across all ANAMMOX species. Despite this shared architecture, ANAMMOX species have diversified through differences in genes, amino acid composition, and physiological activity across environmental conditions. These evolutionary adaptations contribute to the broad genomic and physiological diversity observed among ANAMMOX species today.

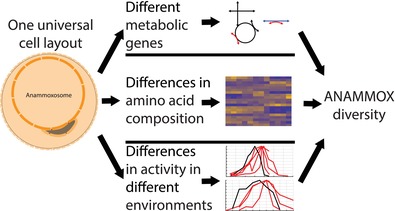

## 1 Introduction

The nitrogen cycle is critically important for all life on earth. All living organisms must either fix nitrogen from the atmosphere or absorb it. For most of human history, we have relied on natural sources of nitrogen such as nitre and guano (Vandermeer 2011). The scarcity and price of these sources prevented their overuse. It also led to a race to produce cheap and plentiful fixed nitrogen compounds to support the agricultural and chemical sector. This led to the invention of the Birkeland–Eyde nitrate production process (Leigh 2004) and later to the Haber‐Bosch ammonia process. With the availability of cheap and plentiful fixed nitrogen, its use as a fertiliser grew rapidly to fuel the agricultural production needed to feed the world's growing population. Now, approximately 80% of the nitrogen present in human tissue samples comes from the Haber‐Bosch process (Howarth 2008). Anthropogenic fixed nitrogen production even outpaces the rest of the biosphere's production, as we produce roughly half of the fixed nitrogen compounds found on earth (Battye et al. 2017). This revolution and independence from nature have not come without its consequences. The fixed nitrogen compounds that we produce have the potential to disrupt ecosystems. Fixed nitrogen is possibly the most important nutrient in regulating primary productivity and species diversity in both aquatic and terrestrial ecosystems (Vitousek et al. 2002). Additionally, biological oxidation of fixed nitrogen to produce nitrogen gas often results in the production of nitrous oxide, resulting in increased greenhouse gas emissions and poorer air quality (Tian et al. 2020).

Returning fixed nitrogen to the atmosphere is generally carried out by a wide range of bacteria, with a few pathways producing the bulk of harmful products. Both nitrification and denitrification produce nitrous oxide as a byproduct (Harris et al. 2021; Liu et al. 2016). Nitrification and denitrification generally occur in environments with abundant oxygen and organic carbon, such as farmland soils, where they are responsible for over 60% of nitrous oxide emissions (Smith et al. 2012). The least polluting way to return nitrogen from fixed nitrogen compounds to the atmosphere is via the ANaerobic AMMonium Oxidation (ANAMMOX) process catalysed by ANAMMOX bacteria. These bacteria produce negligible amounts of nitrous oxide when producing nitrogen gas and are cornerstones of the nitrogen cycle. They are typically responsible for between 10% and 40% of all nitrogen gas production in marine environments (Rios‐Del Toro and Cervantes 2019). Thus, ANAMMOX provides a unique opportunity for us to employ them to meet our own nitrogen oxidation needs in wastewater treatment processes.

ANAMMOX bacteria were first publicly reported in a denitrifying fluidized bed reactor in 1995 (Mulder et al. 1995; van de Graaf et al. 1995). The patents describing their use had been filed already in 1989 and granted in 1992 (Mulder 1992). By 2014 there were over 100 installed ANAMMOX reactors around the world (Lackner et al. 2014). These reactors mostly employed a partial nitration/ANAMMOX configuration which requires approximately half of the ammonium to be converted to nitrite before the ANAMMOX bacteria convert it to nitrogen. This partial aeration stage is not desirable, as it requires significant energy to pump air into the fluid and still produces nitrous oxide. New research into ANAMMOX metabolism has shown that the aeration step is not strictly necessary because the reaction can also be carried out with an electrode acting as the electron acceptor instead of nitrite (Shaw et al. 2020).

It has been known for a while that certain organisms have a consistent bias in the amino acids expressed by their genomes (Kariin and Burge 1995). In bacteria, these biases often relate to amino acid substitutions in enzymes which enable greater activity under new environmental conditions (Fukuchi et al. 2003; Panja et al. 2020; Paul et al. 2008; Raymond‐Bouchard et al. 2018). Those substitutions are often consistent not only at the genus level (Griffiths et al. 2024) but also across different domains of life (Morimoto and Pietras 2024). Halophilic species tend to substitute amino acids in proteins for lower hydrophobicity and higher acidic residues, especially aspartate and the under‐representation of cysteine (Panja et al. 2020). This principle allows us to investigate how the substitution of amino acids has been influenced by the niches that different ANAMMOX bacteria occupy.

The maturity of a process is best expressed in our ability to predict the performance of it under certain conditions. The ANAMMOX process has reached a stage where the behaviour of ANAMMOX bacteria across a range of conditions has been reported not only for generic ANAMMOX sludge but for individual ANAMMOX species. The most consistent and methodical approach has been described in a series of papers detailing the physiological characterisation of different ANAMMOX species (Ali et al. 2015; Awata et al. 2013; Narita et al. 2017; Oshiki et al. 2011). It is immensely valuable for mathematical modelling efforts that the nutrient uptake kinetics, as well as the influence of temperature, pH, and the yield of each species have been consistently reported under similar settings.

This review compiles key discoveries that highlight both the similarities and differences among different ANAMMOX species. We begin with an introduction to the history of ANAMMOX bacteria and the successive developments that have led to the current attention in ANAMMOX technology. We then discuss the physiological characteristics of these bacteria that are common to all ANAMMOX species. This discussion will be followed by our analysis of ANAMMOX diversity in the context of physiological responses to environmental stresses, genetic diversity, genes present in different species, and lastly in terms of the proteomes of different ANAMMOX species. These differences between species are summarised in their relation to the impact they have on industrial and environmental applications.

## 2 Anaerobic Ammonium Oxidation Technology

The first publication mentioning ANAMMOX bacteria was a patent which described a process where ammonia was oxidised in an anoxic reactor (Mulder 1992). It was filed in February of 1989 and included a drawing of the fluidised bed reactor in which the ANAMMOX reaction had been taking place. To explain what was happening inside the reactor, it cited a previous paper which predicted that anaerobic ammonium oxidation ought to be possible (Broda 1977). At this point it was postulated that ANAMMOX bacteria used nitrate as an electron acceptor and ammonium was the electron donor (Kuenen 2008; Mulder et al. 1995). Later it was found that ANAMMOX bacteria preferred nitrite as an electron acceptor and the following chemical equation was established for the respiration of ANAMMOX bacteria:

(1) $${\mathrm{NH}}_{4}^{+}+{\mathrm{NO}}_{2}^{-}\to N_{2}+2H_{2}O$$

The organism responsible for the reaction was then isolated, characterised, and named *Ca. Brocadia anammoxidans* (Strous et al. 1999). The sequencing of the 16S ribosomal RNA has since allowed for the identification of ANAMMOX bacteria, and they have since been found in a variety of anaerobic environments. They have been found in arctic permafrost, farmland soils, lakeshores, marshes, the deepest and shallowest parts of the ocean, wetlands, and mountains (Dalsgaard et al. 2012; Humbert et al. 2012; Humbert et al. 2010; Na et al. 2018; Shen et al. 2016; Wang et al. 2019; Wenk et al. 2013; Xi et al. 2016). When ANAMMOX bacteria were first discovered, it was quite surprising that such a pivotal part of the nitrogen cycle had gone unnoticed until then. Then sulphate reduction coupled to ammonium oxidation was discovered (Fdz‐Polanco 2001), and later nitrite and nitrate dependent oxidation of methane was discovered (Raghoebarsing et al. 2006). The continued discovery of bacteria which make unique contributions to geochemical cycles has led to a renewed interest in the search for elusive, difficult‐to‐culture bacteria denoted ‘spookmicrobes’ (’t Zandt et al. 2020).

**FIGURE 1:**
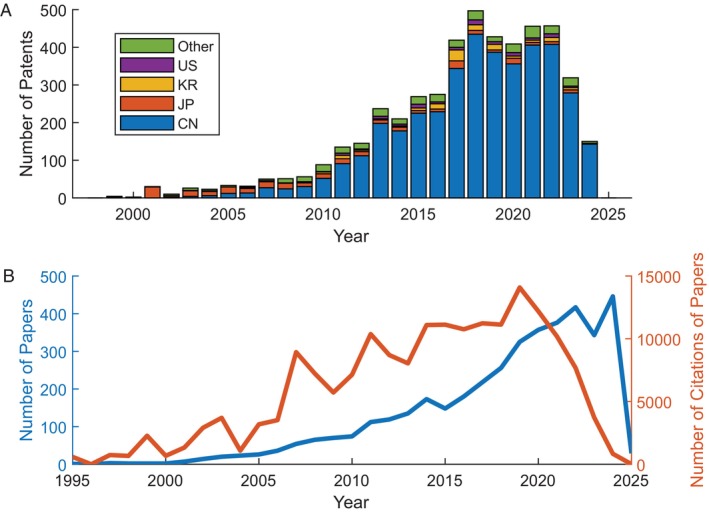
(A) Patents filed containing at least one mention of “ANAMMOX” each year according to Google patents (Google 2024). Number of patents divided by country of submission. The search was made on 05/12/2024. (B) Number of papers mentioning ANAMMOX each year on PubMed (U.S. National Library of Medicine 2025) using the search criteria (“anaerobic ammonium oxidation” [Title/Abstract] OR “anoxic ammonium oxidation” [Title/Abstract] OR ANAMMOX [Title/Abstract]) NOT “fermentation” [Title/Abstract] (left axis). Number of citations for the papers published in that year (right axis). Full methodology is provided in the Supporting Information. CN, China; JP, Japan; KR, South Korea.

For the purposes of this study, we focus only on the bacteria that perform the reaction shown by Equation (1). They are collectively referred to as ANAMMOX bacteria and used industrially to remove fixed nitrogen compounds from wastewater. Commercial and academic interest in ANAMMOX technology has been steadily increasing (Figure 1). The data obtained from carrying out a search for patents mentioning the word ‘ANAMMOX’ is shown in Figure 1A. The largest interest in ANAMMOX technology was found to be in China where the rapid industrialisation has led to an immediate and enhanced need to treat a high concentration of fixed nitrogen‐containing wastewater from the manufacturing sector (Ali et al. 2013). It should be noted that not all these patents were granted and hence, the trend in the figure only provides an indication of commercial interest.

Similarly, the number of academic papers on the topic of ANAMMOX bacteria has also increased over time, as shown in Figure 1B. This figure demonstrates an increased academic interest in these environmentally important bacteria and a growing body of literature on the topic. Citation trends further underscore this growth in knowledge, with earlier seminal papers continuing to influence contemporary research while newer studies address more specific questions regarding the physiology and metabolism of these fascinating bacteria. This rising trend highlights ANAMMOX bacteria as an increasingly popular subject in the area of microbiology and environmental science.

ANAMMOX reactors require less energy to operate and generate substantially less sludge, reducing the need for waste disposal and associated costs (Liu et al. 2021). In theory, ANAMMOX reactors should emit less nitrous oxide. However, this has not been achieved in practice, and the reasons for the observed nitrous oxide emissions have yet to be discovered (Lin et al. 2022). The downsides of ANAMMOX reactors are often stated as slower start‐up times, high nitrate concentration in effluent, and difficulty in achieving stable nitrite to ammonium ratios in their feed (Wang et al. 2022). Currently, start‐up time remains a key area of focus, and relevant discussions are ongoing in ANAMMOX research due to its critical role in determining the feasibility and scalability of the process for industrial applications (Ali and Okabe 2015; Cema et al. 2012; Ganesan and Vadivelu 2019; Lin et al. 2023; Wang et al. 2016).

## 3 Physiological Characteristics of ANAMMOX Bacteria

ANAMMOX bacteria are chemolithoautotrophic prokaryotes that fix inorganic carbon, primarily through the Wood–Ljungdahl pathway (Lawson et al. 2020), and derive energy by oxidising ammonia and reducing nitrite in the anaerobic environment (Strous et al. 1999). All species of ANAMMOX bacteria share similar morphologies and sizes. They are spherical, typically with a diameter of 800–1100 nm (van Niftrik et al. 2008a). They contain a membrane‐bound organelle called the anammoxosome which usually occupies between 66% (van Niftrik et al. 2008a) and 38.9% (Xiong et al. 2024) of the cell volume depending on the species and the salinity in their environment. Different ANAMMOX species are generally indistinguishable in physical appearance, so to identify different species, genomic approaches are used. Collectively, these bacteria render a vibrant reddish‐orange colour as a result of the large number of iron‐containing haem‐proteins (Yin et al. 2023).

**FIGURE 2:**
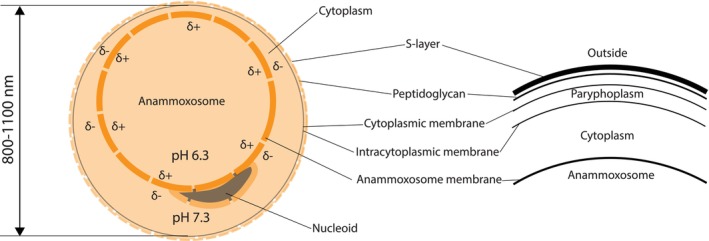
Schematic representation of the ANAMMOX cell structure. Cellular membranes and compartments of a typical ANAMMOX bacterial cell are shown. The pH values are experimental values obtained from literature (van der Star et al. 2010).

ANAMMOX bacteria have a unique cell plan (Figure 2) that divides their cytoplasm into three separate compartments (van Niftrik et al. 2008b). Starting from the outermost layer, they have an S‐layer composed of a crystalline array of protein subunits (van Teeseling et al. 2014). Next, they have a thin layer of peptidoglycan (Van Teeseling et al. 2015), followed by the cytoplasmic and intracytoplasmic membranes, which are composed of fatty acids and ethers with ladderane moieties (linearly concatenated cyclobutane rings) (Sinninghe Damsté et al. 2005; Sinninghe Damsté et al. 2002). The nucleoid, cytoplasm, and anammoxosome comprise the rest of the cell (Figure 2). The anammoxosome is a unique organelle to ANAMMOX bacteria, and its membrane is highly enriched in ladderanes (Neumann et al. 2014).

The S‐layer or the surface layer is a highly organised crystalline structure composed of proteins or glycoproteins that forms the outermost component of the cell envelope in ANAMMOX bacteria. It consists of hexagonal units that entirely cover the cell's surface, each made up of six protein subunits with a total molecular mass of 160 kDa. These proteins exhibit no homology to any previously characterised proteins (van Teeseling et al. 2014). The S‐layer in ANAMMOX bacteria plays a critical role in biofilm formation and interactions, helping to curate the microbial community within the biofilm. ANAMMOX bacteria secrete extracellular polymeric substances (EPS), which include S‐layer glycoproteins, to construct and maintain these biofilms (Li Wong et al. 2023). The mechanical and rheological properties of ANAMMOX EPS have been investigated (Lotti et al. 2019) and are currently being explored for potential applications, such as for coating paper to make it more water and grease resistant (Feng et al. 2024).

For a long time, members of the phylum *Planctomycetota* were thought to lack peptidoglycan entirely. However, the presence of peptidoglycan was first identified in other members of this group, including *Planctomyces limnophilus* , *Gemmata obscuriglobus* , and *Rhodopirellula baltica* (Jeske et al. 2015) before being confirmed in ANAMMOX bacteria (Van Teeseling et al. 2015). Unlike the thick, rigid peptidoglycan found in many bacteria, the layer in ANAMMOX bacteria is notably thin and often chemically modified. These modifications include unique cross‐linkages that may enhance their ability to withstand the anaerobic, high‐pressure environments in which they thrive. Despite its reduced thickness, the peptidoglycan layer plays a critical role in maintaining structural integrity and cellular shape. Furthermore, its distinctive structure could contribute to natural antibiotic resistance, making it an important area of study for understanding both bacterial evolution and potential biotechnological applications (Van Teeseling et al. 2015).

The cytoplasmic, intracytoplasmic, and anammoxosome membranes of ANAMMOX bacteria contain ladderane lipids, which are thought to be unique to this group (van Niftrik et al. 2008b). The highest concentration of ladderanes is found in the anammoxosome membrane (Neumann et al. 2014). These lipids exhibit several distinctive properties, including low permeability to protons and hydroxide ions, high packing density, and surprisingly high fluidity despite their rigid cyclobutane structure (Boumann et al. 2009; Moss et al. 2018; Sinninghe Damsté et al. 2002). Originally, ladderanes were thought to protect the cell from leakage of toxic intermediates such as hydrazine; however, this hypothesis has been shown to be inaccurate by later investigation (Moss et al. 2018).

The anammoxosome is a specialised compartment unique to ANAMMOX bacteria and is the site of catabolism and nitrogen production (de Almeida et al. 2015; Kartal and Keltjens 2016). It is also the primary site of ATP synthesis since there is a pH gradient across its membrane (van der Star et al. 2010). The compartmentalisation provided by the anammoxosome membrane ensures that the highly reactive intermediates involved in the ANAMMOX process remain relatively contained, protecting the rest of the cell from potential damage. This organelle also demonstrates a degree of structural flexibility, as its size can vary in response to environmental factors, such as changes in salinity (Xiong et al. 2024). Ongoing research continues to uncover additional components and functions of this organelle. For instance, encapsulation proteins within the anammoxosome have been identified to mitigate chemical stress from hydroxylamine (Xing et al. 2020), and iron nanoparticles have been discovered, which both reduce iron toxicity and serve as a storage mechanism (Peng et al. 2022).

ANAMMOX bacteria change their physiology in response to environmental stresses. The genus *Scalindua* can change the size and morphology of the anammoxosome in order to protect themselves from higher salinity (Xiong et al. 2024). ANAMMOX bacteria also alter the ladderane composition of their lipid membranes in response to temperature (Rattray et al. 2010). These differences in adaptability in different ANAMMOX species can be attributed to genetic differences.

## 4 Distribution of ANAMMOX Species in a Community

Different ANAMMOX species have different temperature, pH, and salinity tolerances. These differences lead to different community compositions in different ANAMMOX reactors and habitats. Determining what causes a specific species to flourish in an environment is a subject of ongoing investigation (Zhang and Okabe 2020). Creating a comprehensive theoretical framework of what determines the dominant ANAMMOX species in a location is also hampered by their complex interactions with other bacteria (Lawson et al. 2017). Since different ANAMMOX species have genes enabling them to utilise alternative energy or carbon sources, there is interest in controlling the microbial community in an ANAMMOX reactor. ANAMMOX reactors are often dominated by a single ANAMMOX species alongside a diverse community of other microorganisms, typically belonging to the phyla *Proteobacteria* or *Chloroflexi* (Pereira et al. 2017).

In natural settings, it is difficult to predict the abundance of different ANAMMOX species. A model developed using statistical methods, co‐occurrence of species, environmental factors, and location found little success predicting the community composition of ANAMMOX bacteria (Wang et al. 2019). It was found that ANAMMOX bacteria were not competing for substrates (ammonium and nitrite) and that substrate availability was not a determining factor for ANAMMOX community composition. Instead, environmental pressures such as oxygen exposure and unstable habitat conditions may play more vital roles (Wang et al. 2019). Oxygen exposure has since also been found to determine ANAMMOX species distribution in aquifers (Mosley et al. 2022).

## 5 Influence of pH and Temperature on the Metabolic Activity of ANAMMOX Bacteria

**FIGURE 3:**
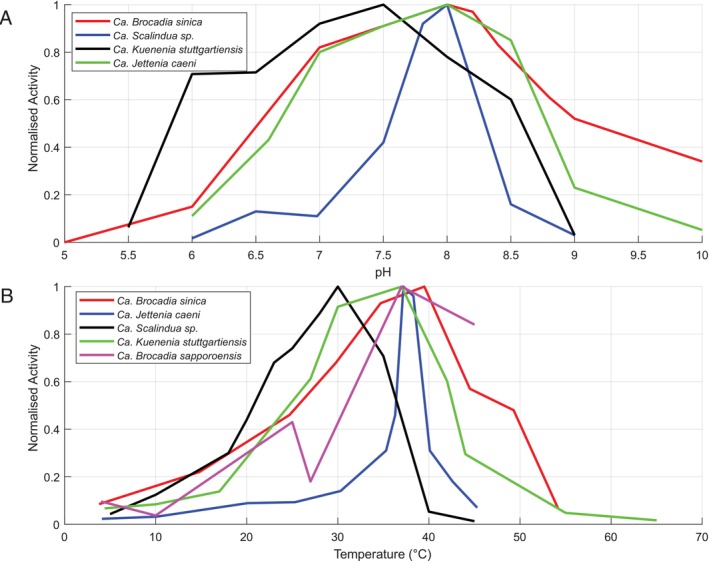
Normalised activity profile of different ANAMMOX species. The normalised activity of different ANAMMOX species with respect to various pH and temperatures is shown in (A) and (B), respectively. The normalised activities were calculated from the reported SAA and absolute ANAMMOX activity data (Ali et al. 2015; Awata et al. 2013; Narita et al. 2017; Oshiki et al. 2011; van der Star et al. 2008), and the values reported in each study are presented in Tables S1–S5.

Specific ANAMMOX Activity (SAA) is a widely used benchmark for assessing the nitrogen oxidation potential of a sample containing ANAMMOX bacteria (Yu et al. 2022). It is often measured under varying environmental conditions such as temperature, pH, or salinity to evaluate how these factors influence the respiratory capabilities of the cells. Unfortunately, the measurement of SAA of different species is generally carried out with incomparable units, which makes it difficult to compare them directly. For example, the activity of *Candidatus Brocadia sinica* under different temperatures has been reported with units of $\mathrm{kg}\;N\;m^{-3}\;d^{-1}$ (Oshiki et al. 2011), whereas for *Candidatus Brocadia sapporiensis*, the SAA is reported as a percentage of the highest activity recorded for the sample (Narita et al. 2017). To reconcile these measurements and compare them side by side across different ANAMMOX species, we converted SAA values to normalised activity values (Figure 3). The normalised activity values were calculated by dividing each SAA value by the highest recorded value for each species and plotted against different pH (Figure 3A) and temperature (Figure 3B) conditions.

It is apparent that different ANAMMOX species do in fact have different sensitivities and ranges of values under which they exhibit their highest nitrogen removal rates. This level of resolution of activity data has important applications such as for simulation of ANAMMOX nitrogen removal rates. The range of values for which a species can tolerate without losing substantial performance is an important consideration when designing a reactor controller. It determines the tolerances that each controlled parameter can deviate from the setpoint. For most reported ANAMMOX bacteria, their tolerance is reported only as a range. For example, *Ca. Brocadia* sp. 40 is reported to grow within a pH range of 7.2–8.3 (Lotti et al. 2014). While such ranges provide a general understanding of the organism's tolerance, it lacks the resolution necessary to design a pH controller with appropriate sensitivity to ensure high performance. The values used to generate Figure 3 are available in Tables S1–S5.

An alternative niche‐based classification of ANAMMOX species differentiates between rapidly growing taxa and those adapted to nutrient‐scarce environments as scavengers. This is best expressed in the Monod growth kinetics parameters with respect to ammonium and nitrite (Zhang and Okabe 2020). To truly understand the dynamics that influence the community composition will require consideration of far more variables such as the interactions of each species with organic matter, inhibition, starvation kinetics, salinity, temperature, and pH. By considering all possible environmental variables, it may be possible to elucidate the factors that influence ANAMMOX community dynamics.

## 6 Genetic Signatures of Environmental Adaptations in ANAMMOX Bacteria

ANAMMOX bacteria are an ancient and phylogenetically deep‐branching group of microorganisms. Phylogenomic and molecular clock analyses suggest that their last common ancestor lived approximately 2.1 billion years ago (Liao et al. 2022), indicating that ANAMMOX bacteria have persisted through a substantial portion of Earth's geological history. Over this immense timescale, they have diversified into a wide range of distinct species, each adapted to specific ecological niches and environmental conditions.

This evolutionary diversification is reflected in genomic content, which varies substantially among ANAMMOX species. The full scope of this genetic variability can be captured through the construction of a pangenome, a collective genome that encompasses all genes present within a defined group of organisms. The pangenome typically consists of a core genome, shared across all members of the group, and an accessory genome comprising genes found only in a subset of taxa, and a unique genome which contains only the genes found in a single species (Tettelin and Medini 2020).

A pangenome of ANAMMOX bacteria has been produced from 12 different species of ANAMMOX bacteria. It was found that only a small proportion of their genes (8.1%) is common to all ANAMMOX bacteria (Bielski and Islam 2024). In the present study, the dispensable genes identified in their analysis were used as the basis for principal component analysis (PCA) to explore patterns of functional divergence and ecological adaptation.

**FIGURE 4:**
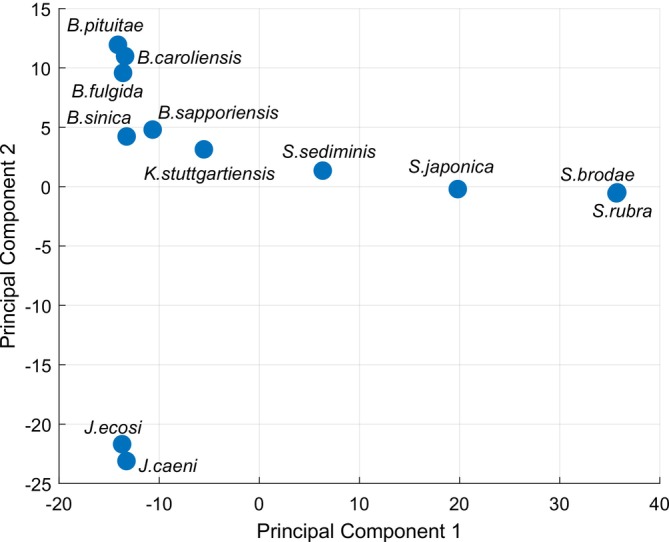
Principal component analysis (PCA) of the dispensable genes in the ANAMMOX pangenome (Bielski and Islam 2024). Methods to perform PCA are provided in the Supporting Information.

Figure 4 shows the PCA of the ANAMMOX pangenome. It demonstrates significant co‐variance in genes associated with environmental adaptations. In principal component 1, there is a clear separation between the halophilic *Scalindua* genus and the non‐halophilic *Brocadia* and *Jettenia* genera, with the halotolerant *Ca. Kuenenia stuttgartiensis* bridging the two groups. Principal component 2 primarily separates the *Jettenia* genus from the others, reflecting additional genetic differences unrelated to salinity tolerance.

The first 2 principal components represent 53% of the variance (39.8% and 13.3%). Most genes with high coefficients related to principal component 1 were not related to osmotic stress but rather a mix of metabolic, non‐metabolic, and hypothetical genes. There were notable genes related to sodium transport which had high coefficients such as Na(+), Li(+), K(+)/H(+) antiporter subunits A and B (coefficient of 0.022) and Na(+)/H(+) antiporter with all its subunits (coefficient of 0.017) with respect to principal component 1. The coefficients of covariance also revealed various metabolic functions which appear to be prevalent only in the *Scalindua* genus of ANAMMOX bacteria, such as the sodium/glucose cotransporter. Principal component 2 represents a smaller proportion of the variance and contains a higher number of hypothetical proteins.

**FIGURE 5:**
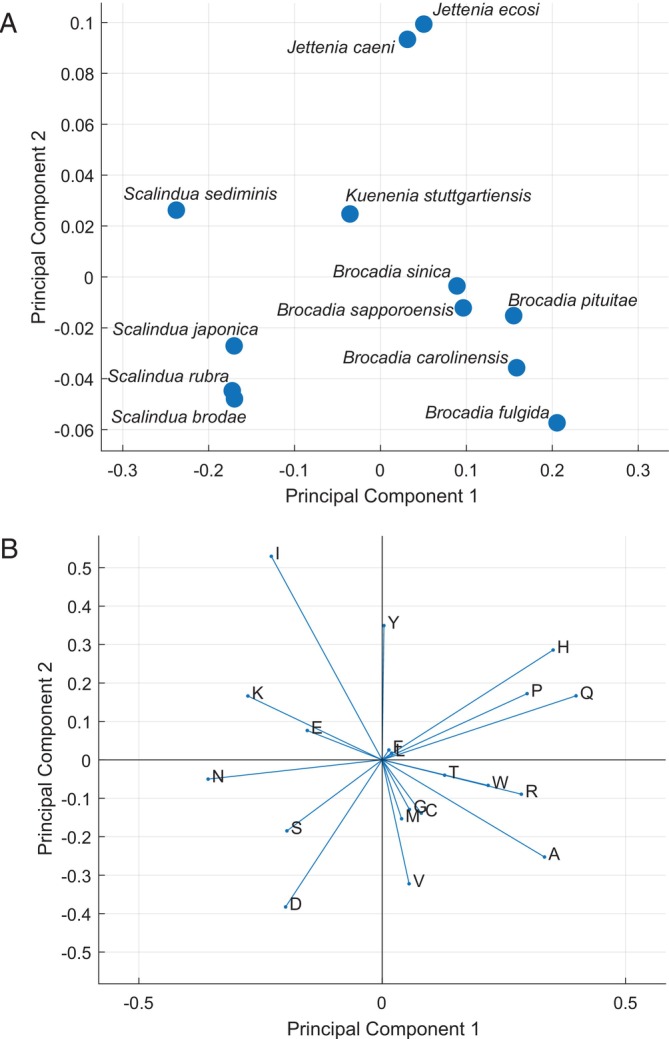
Principal component analysis (PCA) of amino acid proportions derived from genomic data of ANAMMOX bacteria. (A) PCA plot showing clustering of ANAMMOX species based on the amino acid composition encoded by their genomes. (B) PCA coefficients illustrating the contributions of individual amino acids to the variance captured by the first two principal components (principal component 1 and principal component 2). Methods used to perform PCA and the genomic data used are provided in Tables S6 and S7.

PCA can also be performed with the amino acid composition of each ANAMMOX genome, and a similar pattern emerges as shown in Figure 5. In this case, the adaptation to salinity was identified to be the primary reason for the differences in amino acid composition. The specific amino acids which are enriched in the *Scalindua* genus (Figure 5A) correspond to previously reported amino acid profiles, enabling a greater enzymatic function in saline environments (Panja et al. 2020; Paul et al. 2008).

If environmental conditions are the primary drivers of genome‐wide amino acid substitutions, then Figure 5A,B offer valuable insights into the probable activity profiles of uncharacterized ANAMMOX bacteria. Principal component 1 appears to correspond to halotolerance, and principal component 2 may be indicative of another adaptation. Principal component 2 does not appear to be correlated to pH or temperature preference of the species examined here. Expanding the dataset with experimentally determined activity profiles across a wider range of environmental conditions would help confirm whether these genomic patterns reliably predict physiological adaptations, or if they are coincidental and not indicating anything substantive. This analysis would not only enhance our understanding of ANAMMOX diversity but also improve the quality of predictive models for their ecological distribution and biotechnological applications.

## 7 Conclusion

ANAMMOX bacteria have successfully adapted to a diverse range of environments, allowing them to occupy their specialised metabolic niche across various ecological and industrial settings. This adaptation has driven substantial genetic variation among ANAMMOX species, reflected in their distinct genomic and physiological characteristics. Over evolutionary timescales, these bacteria have acquired numerous metabolic and non‐metabolic genes through both natural selection and genetic drift, contributing to their functional diversity. The most significant evolutionary pressure influencing the ANAMMOX pangenome is salinity tolerance, which significantly shapes genetic differentiation between halophilic and non‐halophilic species. Comparative genomic analyses reveal that halophilic ANAMMOX bacteria possess a unique set of genes and exhibit distinct trends in amino acid composition, particularly a shift toward more acidic amino acids. These adaptations suggest that environmental pressures, rather than evolutionary drift, have been the dominant force in shaping ANAMMOX diversity. Adaptations to different temperature and pH have not altered the genomes of ANAMMOX bacteria in ways which can be easily determined from examination of their genomes.

The ability of ANAMMOX bacteria to maintain metabolic function under diverse conditions has direct implications for their ecological roles and applications in engineered systems, particularly for wastewater treatment processes. A deeper understanding of their physiological responses to environmental stresses is essential for optimising their performance in biotechnological applications and predicting their distribution in the natural environment. Future research should focus on integrating genomic, proteomic, and physiological data to refine models of ANAMMOX community dynamics and improve the efficiency of ANAMMOX‐based nitrogen removal processes.

## Author Contributions

**Roman G. Bielski:** conceptualization (lead), data curation (lead), formal analysis (lead), methodology (equal), project administration (supporting), visualization (lead), writing – original draft (lead), writing – review and editing (equal). **Dr. M. Ahsanul Islam:** conceptualization (supporting), data curation (supporting), formal analysis (supporting), methodology (equal), project administration (lead), visualization (supporting), writing – original draft (supporting), writing – review and editing (equal).

## Conflicts of Interest

The authors declare no conflicts of interest.

## Supporting information


**Data S1:** Supporting Information.

## Data Availability

The data that support the findings of this study are available in PubMed at https://pubmed.ncbi.nlm.nih.gov/. These data were derived from the following resources available in the public domain: https://pubmed.ncbi.nlm.nih.gov/, https://pubmed.ncbi.nlm.nih.gov/.
