# Supplementary material for: Genomic and Physiological Signatures of Evolution in ANAMMOX Bacteria
Source: Environ Microbiol Rep. 2025 Sep 18;17(5):e70197. doi: 10.1111/1758-2229.70197 (PMC12445922; doi:10.1111/1758-2229.70197)
Supplement: Supplementary file 1 — Data S1: Supporting Information. [file EMI4-17-e70197-s001.docx]

**Supplementary File**

**Genomic and Physiological Signatures of Evolution in ANAMMOX Bacteria**

Roman G. Bielski and M. Ahsanul Islam*

Department of Chemical Engineering, Loughborough University, Loughborough, Leicestershire, LE11 3TU

*Corresponding author: m.islam@lboro.ac.uk

Running title: Evolutionary Signatures in ANAMMOX Bacteria

Figure creation methodology

Figure 1

Figure 1 is derived from a google patents search with the keyword “ANAMMOX”. These results were sorted by date and country of submission. The search was made on 05/12/2024.

The search for papers on PubMed related to ANAMMOX bacteria was performed by inserting the following into the PubMed search bar:

("anaerobic ammonium oxidation"[Title/Abstract] OR "anoxic ammonium oxidation"[Title/Abstract] OR ANAMMOX[Title/Abstract]) NOT "fermentation"[Title/Abstract]

The Digital Object Identifiers (DOIs) were then used in a python script to find the number of citations each paper had with the crossref.org API. The number of citations for the papers published in that year are the right-hand axes in the figure. The figure was generated in MATLAB with the following code:

%combined citation and patent histogram

%histogram of patents containing ANAMMOX references

data1 = [0 1998

4 1999

2 2000

30 2001

10 2002

26 2003

23 2004

33 2005

31 2006

50 2007

51 2008

56 2009

88 2010

135 2011

145 2012

237 2013

210 2014

269 2015

275 2016

419 2017

497 2018

428 2019

409 2020

456 2021

457 2022

319 2023

150 2024

0 2025];

countries_patents = [0 0 0 0

1 3 4 4

0 0 2 2

0 29 29 29

2 6 6 6

4 19 20 20

6 17 19 19

12 29 29 29

13 25 28 28

27 43 46 46

24 39 40 40

30 40 43 43

52 64 69 70

91 104 113 119

112 123 127 130

198 207 210 217

178 188 191 196

225 232 239 249

229 236 250 255

344 364 393 400

435 445 460 473

387 393 408 415

356 371 377 386

406 413 419 425

408 415 426 436

279 287 293 297

143 144 144 145

0 0 0 0];

%different sums needed for bar chart so other is used instead of total for

%final part

countries_bar = [0 0 0 0 0

1 2 1 0 0

0 0 2 0 0

0 29 0 0 1

2 4 0 0 4

4 15 1 0 6

6 11 2 0 4

12 17 0 0 4

13 12 3 0 3

27 16 3 0 4

24 15 1 0 11

30 10 3 0 13

52 12 5 1 18

91 13 9 6 16

112 11 4 3 15

198 9 3 7 20

178 10 3 5 14

225 7 7 10 20

229 7 14 5 20

344 20 29 7 19

435 10 15 13 24

387 6 15 7 13

356 15 6 9 23

406 7 6 6 31

408 7 11 10 21

279 8 6 4 22

143 1 0 1 5

0 0 0 0 0];

%histogram of ANAMMOX related papers on Pubmed

data = [1 1995

0 1996

3 1997

2 1998

2 1999

2 2000

7 2001

14 2002

20 2003

23 2004

26 2005

36 2006

54 2007

65 2008

70 2009

74 2010

112 2011

119 2012

135 2013

173 2014

148 2015

180 2016

218 2017

256 2018

325 2019

357 2020

376 2021

417 2022

343 2023

446 2024

29 2025];

citations = [1995 586

1996 0

1997 734

1998 666

1999 2271

2000 672

2001 1330

2002 2907

2003 3697

2004 1083

2005 3183

2006 3517

2007 8933

2008 7233

2009 5720

2010 7135

2011 10372

2012 8703

2013 8043

2014 11095

2015 11123

2016 10756

2017 11232

2018 11129

2019 14086

2020 12193

2021 10189

2022 7680

2023 3727

2024 834

2025 9];

tiledlayout(2,1)

% First tile (top panel)

nexttile

hold on

bar(data1(:,2),countries_bar,'stacked');

legend('CN','JP','KR','US','Other');

xlabel('Year');

ylabel('Number of Patents');

hold off

% Second tile (bottom panel)

nexttile

hold on

yyaxis left

plot(data(:,2),data(:,1),"LineWidth",2);

xlabel('Year');

ylabel('Number of Papers');

yyaxis right

plot(citations(:,1),citations(:,2),"LineWidth",2)

ylabel('Number of Citations of Papers')

hold off

The figure was then edited in Adobe Illustrator (Adobe Inc. 2019) to include the A and B to distinguish the panels.

Figure 2

This was drawn in Adobe Illustrator (Adobe Inc. 2019). The style was inspired by the original drawing of the structural layers in an ANAMMOX bacterium (Van Niftrik et al. 2008) but updated to include more recently discovered structural elements.

Figures 3

These pH and temperature activity data were gathered from a variety of publications in which the raw data had not been provided. Therefore, these data were extracted manually from figures. Normalized activity values were calculated by dividing the specific ANAMMOX activity value by the highest recorded value for each species.

Table 1: Activity parameters for “Ca. Brocadia sinica” across a range of pH and temperatures. Data extracted from (Oshiki et al. 2011).

| pH | Normalised Activity | Temperature | Normalised Activity |
| --- | --- | --- | --- |
| 5 | 0 | 3.833333 | 0.085427 |
| 6 | 0.147783 | 14.83333 | 0.21608 |
| 7 | 0.82266 | 24.66667 | 0.462312 |
| 8 | 1 | 29.83333 | 0.678392 |
| 8.2 | 0.970443 | 34.66667 | 0.934673 |
| 8.4 | 0.832512 | 39.5 | 1 |
| 8.6 | 0.724138 | 44.5 | 0.572864 |
| 8.8 | 0.610837 | 49.33333 | 0.482412 |
| 9 | 0.517241 | 54.16667 | 0.065327 |
| 10 | 0.339901 |  |  |

Table 2: Activity parameters for “Ca. Jettenia caeni” across a range of pH and temperatures. Data extracted from (Ali et al. 2015).

| Temperature | Normalised Activity | pH | Normalised Activity |
| --- | --- | --- | --- |
| 4.078947 | 0.023474 | 6 | 0.016949 |
| 10.13158 | 0.032864 | 6.487179 | 0.127119 |
| 20.13158 | 0.089202 | 6.987179 | 0.114407 |
| 25.26316 | 0.093897 | 7.5 | 0.419492 |
| 30.26316 | 0.140845 | 7.807692 | 0.923729 |
| 35.26316 | 0.305164 | 8 | 1 |
| 36.31579 | 0.464789 | 8.5 | 0.161017 |
| 37.23684 | 1 | 9 | 0.029661 |
| 38.28947 | 0.957746 |  |  |
| 40.13158 | 0.314554 |  |  |
| 42.63158 | 0.183099 |  |  |
| 45.26316 | 0.065728 |  |  |

Table 3: Activity parameters for “Ca. Brocadia sapporiensis” across a range of pH and temperatures. Data extracted from (Narita et al. 2017).

| Temperature | Normalised Activity |
| --- | --- |
| 10.01575 | 0.283186 |
| 19.9685 | 0.544248 |
| 24.94488 | 0.59292 |
| 29.92126 | 0.69469 |
| 36.91339 | 1 |
| 44.91339 | 0.681416 |

Table 4: Activity parameters for “Ca. Scalindua sp.” across a range of pH and temperatures. Data extracted from (Awata et al. 2013).

| Temperature | Normalised Activity | pH | Normalised Activity |
| --- | --- | --- | --- |
| 4.964029 | 0.043189 | 5.492958 | 0.063063 |
| 10.07194 | 0.124585 | 5.992958 | 0.708108 |
| 14.89209 | 0.303987 | 6.492958 | 0.715315 |
| 18.05755 | 0.44186 | 6.985915 | 0.924324 |
| 20 | 0.679402 | 7.485915 | 1 |
| 23.09353 | 0.742525 | 7.978873 | 0.783784 |
| 24.96403 | 0.887043 | 8.485915 | 0.601802 |
| 28.05755 | 1 | 8.978873 | 0.032432 |
| 30.14388 | 0.707641 |  |  |
| 35.2518 | 0.053156 |  |  |
| 40.35971 | 0.013289 |  |  |
| 45.10791 | 0 |  |  |

Table 5: Activity parameters for “Ca. Kuenenia stuttgartiensis” across a range of pH and temperatures. (Van Der Star et al. 2008).

| Temperature | Normalised Activity | pH | Normalised Activity |
| --- | --- | --- | --- |
| 4.4 | 0.067189 | 6 | 0.11138 |
| 10 | 0.083986 | 6.6 | 0.431268 |
| 17 | 0.137583 | 7 | 0.801887 |
| 17 | 0.613545 | 7.5 | 0.905746 |
| 30 | 0.915123 | 8 | 1 |
| 37 | 1 | 8.5 | 0.849577 |
| 42 | 0.602445 | 9 | 0.237324 |
| 44 | 0.295198 | 10 | 0.05169 |
| 55 | 0.047632 |  |  |
| 65 | 0.016797 |  |  |

Figure 4

The disposable genome from a previously published pangenome (Bielski and Islam 2024) of the same 12 ANAMMOX annotated genomes was used to perform PCA analysis in MATLAB. First, the dispensable genes were prepared in a CSV file by taking the dispensable genome sheet from the supplementary files (Bielski and Islam 2024), replacing all the blank cells with “0” and replacing the gene references with “1” . This file’s location should then be placed in the “Your file location” section at the beginning of the following MATLAB code:

% Specify the file path

filepath = 'C:\your_file_location\formatted_data.csv';

% Load the table and preserve original column headers

dataTable = readtable(filepath, 'VariableNamingRule', 'preserve');

% Extract gene names (first column)

geneNames = dataTable{:, 1}; % First column: gene names

% Extract species data (remaining columns)

speciesData = dataTable{:, 2:end}; % All other columns: binary presence/absence

% Convert speciesData to numeric

if iscell(speciesData)

speciesData = str2double(speciesData); % Convert cell array to numeric array

end

% Get species names from the column headers

speciesNames = dataTable.Properties.VariableNames(2:end); % Column headers for species

% Check the dimensions of the data

disp('Dimensions of speciesData:');

disp(size(speciesData));

% Perform PCA (transpose so species are rows)

try

[coeff, score, ~] = pca(speciesData');

catch ME

disp('Error during PCA:');

disp(ME.message);

return;

end

% Plot the first two principal components

figure;

scatter(score(:, 1), score(:, 2), 100, 'filled'); % Scatter plot with large points

text(score(:, 1), score(:, 2), speciesNames, 'VerticalAlignment', 'bottom', 'HorizontalAlignment', 'right');

title('PCA of Dispensable Genome Similarity');

xlabel('Principal Component 1');

ylabel('Principal Component 2');

grid on;

% Perform PCA (transpose so species are rows)

[coeff, score, latent, tsquared, explained] = pca(speciesData');

% Scree Plot - Variance Explained by Each Principal Component

figure;

bar(explained, 'FaceColor', [0.2, 0.6, 0.8]); % Bar plot with custom color

xlabel('Principal Component');

ylabel('Percentage of Variance Explained');

title('Scree Plot');

grid on;

% Choose the principal components to plot (e.g., PC1 vs PC3)

pcX = 2; % Principal Component for X-axis

pcY = 3; % Principal Component for Y-axis

% Scatter plot of the chosen principal components

figure;

scatter(score(:, pcX), score(:, pcY), 100, 'filled'); % Scatter plot with large points

text(score(:, pcX), score(:, pcY), speciesNames, 'VerticalAlignment', 'bottom', 'HorizontalAlignment', 'right');

title(sprintf('PCA Plot: PC%d vs PC%d', pcX, pcY));

xlabel(sprintf('Principal Component %d', pcX));

ylabel(sprintf('Principal Component %d', pcY));

grid on;

% Sort loadings for PC1

[sortedLoadings, idx] = sort(abs(coeff(:, 2)), 'descend');

% Display top 10 genes contributing to PC1

topGenes_PC1 = geneNames(idx(1:1500)); % geneNames is the list of gene labels

topLoadings_PC1 = coeff(idx(1:1500), 2); % Corresponding loadings

disp(table(topGenes_PC1, topLoadings_PC1));

This code should be run in MATLAB (The MathWorks Inc. 2022), and it will generate the relevant figures and display the genes with the highest coefficients, as well as their annotated functions.

Figure 5

The genome sequences for 12 ANAMMOX species were downloaded form NCBI’s database. The protein sequences were used to determine the total number of times each amino acid was expressed in the proteome. This was normalized and used as the input to the PCA function in MATLAB then the coefficients were plotted alongside it in the biplot.

Table 6: Number of each amino acid in each genome file (A-L) and Genbank or RefSeq ID.

| Genome file (Genbank ID) | A | C | D | E | F | G | H | I | K | L |
| --- | --- | --- | --- | --- | --- | --- | --- | --- | --- | --- |
| *Brocadia carolinensis* GCA_002009475.1 | 61554 | 12047 | 45760 | 58499 | 39775 | 60547 | 19764 | 67799 | 63100 | 81686 |
| *Brocadia fulgida* GCA_000987375.1 | 70980 | 13552 | 51607 | 65505 | 43824 | 68601 | 22325 | 74878 | 68344 | 91635 |
| *Brocadia pituitae* GCA_017347445.1 | 77521 | 14535 | 58828 | 74769 | 49176 | 76823 | 25309 | 85211 | 79638 | 102636 |
| *Brocadia sapporoensis* GCF_001753675.2 | 55162 | 10564 | 41521 | 53379 | 35258 | 54674 | 17168 | 63721 | 57680 | 73225 |
| *Brocadia sinica* GCA_000949635.1 | 76423 | 15020 | 58933 | 76061 | 50891 | 79011 | 24474 | 90500 | 82231 | 105384 |
| *Jettenia caeni* GCF_000296795.1 | 70954 | 14292 | 57510 | 74994 | 49409 | 74495 | 24560 | 94479 | 81238 | 102243 |
| *Jettenia ecosi* GCA_005524015.1 | 68143 | 13605 | 53871 | 71549 | 47214 | 70682 | 23791 | 89309 | 77318 | 97467 |
| *Kuenenia stuttgartiensis* GCF_900232105.1 | 74632 | 15092 | 56550 | 76915 | 49745 | 74282 | 23320 | 90488 | 83431 | 102398 |
| *Scalindua brodae* GCA_000786775.1 | 71106 | 14731 | 64263 | 78776 | 50549 | 76598 | 22524 | 94054 | 85897 | 104450 |
| *Scalindua japonica* GCF_002443295.1 | 79735 | 17121 | 73814 | 90964 | 58666 | 88981 | 26715 | 108338 | 100769 | 120607 |
| *Scalindua rubra* GCA_002632345.1 | 72005 | 14662 | 64956 | 79470 | 50978 | 77585 | 22705 | 95298 | 86549 | 105339 |
| *Scalindua sediminis* GCA_017368835.1 | 52930 | 11296 | 46700 | 62125 | 37624 | 58109 | 16660 | 75023 | 71182 | 78823 |

Table 7: Number of each amino acid in each genome file (M-Y)

| Genome file | M | N | P | Q | R | S | T | V | W | Y |
| --- | --- | --- | --- | --- | --- | --- | --- | --- | --- | --- |
| *Brocadia carolinensis* | 21697 | 37378 | 34870 | 28654 | 44953 | 52915 | 48309 | 59560 | 9653 | 30057 |
| *Brocadia fulgida* | 24457 | 40573 | 39641 | 31678 | 52581 | 58310 | 52927 | 66168 | 10656 | 33372 |
| *Brocadia pituitae* | 26844 | 47268 | 43881 | 36167 | 56809 | 65638 | 61128 | 73746 | 12047 | 38626 |
| *Brocadia sapporoensis* | 19537 | 33763 | 31163 | 25393 | 39948 | 47307 | 43097 | 53240 | 8157 | 27242 |
| *Brocadia sinica* | 27179 | 49443 | 44710 | 35578 | 56687 | 67264 | 61848 | 75721 | 12533 | 39451 |
| *Jettenia caeni* | 26186 | 49016 | 43352 | 35062 | 53649 | 66986 | 59570 | 70261 | 11826 | 39564 |
| *Jettenia ecosi* | 25529 | 46022 | 41401 | 33503 | 51385 | 63076 | 55588 | 66656 | 10946 | 37302 |
| *Kuenenia stuttgartiensis* | 26838 | 51246 | 40937 | 32905 | 52287 | 67167 | 58871 | 70395 | 11530 | 39585 |
| *Scalindua brodae* | 27931 | 54039 | 40745 | 32238 | 52047 | 72717 | 59529 | 74239 | 11478 | 38520 |
| *Scalindua japonica* | 31401 | 64744 | 47183 | 38424 | 60909 | 85619 | 68664 | 86181 | 13786 | 45262 |
| *Scalindua rubra* | 27682 | 54320 | 41066 | 32385 | 52341 | 73386 | 60142 | 74888 | 11584 | 38794 |
| *Scalindua sediminis* | 20400 | 40107 | 30597 | 22959 | 40942 | 52614 | 43646 | 56086 | 8121 | 29739 |

The figures in the main text can be generated from the following MATLAB code which contains the normalized amino acid data to facilitate loading:

amino_acid_proportions = [1.05507774 1.08893827 1.054961742 1.049934687 1.01907609 0.971699534 0.982607958 1.023029037 0.950625948 0.918096039 0.954422624 0.93153033

1.032654567 1.039725364 0.989191464 1.005538382 1.00161413 0.978804818 0.981082556 1.034564703 0.984880696 0.985862216 0.971894816 0.994186289

0.971911856 0.981044175 0.992005572 0.979270719 0.97376411 0.975913134 0.962557648 0.960523897 1.064577041 1.053150746 1.066866376 1.018414726

0.969134846 0.971290865 0.983436908 0.981974705 0.980284528 0.99263416 0.997172248 1.019017194 1.017899563 1.012316351 1.018096328 1.056742307

1.009839511 0.995848495 0.991253045 0.994017302 1.005165341 1.002247925 1.008425488 1.010011471 1.000990544 1.000551813 1.000863506 0.980785558

1.005371517 1.019538147 1.012778269 1.00811226 1.020647688 0.988298197 0.987355671 0.986398122 0.992034129 0.992526623 0.996234558 0.990704818

1.047529679 1.059063607 1.065014795 1.010427822 1.00913855 1.040032198 1.060803724 0.988450073 0.93113413 0.951168818 0.930599657 0.906636948

0.942225328 0.931375497 0.940191482 0.983350106 0.978440488 1.049044002 1.04413718 1.005673893 1.019492399 1.011400665 1.024153759 1.070515201

0.962262062 0.932832103 0.964214178 0.976749856 0.975559867 0.989806173 0.991917297 1.017480639 1.021685515 1.032290399 1.020647907 1.114554004

1.001325292 1.005374871 0.998887177 0.996738047 1.004978069 1.001355 1.005115574 1.003815115 0.998645239 0.993141216 0.998542693 0.992081707

1.01375854 1.022767941 0.995797957 1.013647173 0.987922008 0.977531883 1.003458666 1.002812378 1.017879945 0.985574029 1.000189272 0.978660208

0.944557558 0.917674312 0.94835024 0.947430795 0.972011788 0.98963983 0.978382562 1.035635767 1.065111092 1.099063614 1.061504941 1.0406375

1.037214331 1.055359075 1.036292229 1.029318838 1.034607402 1.030273739 1.035996385 0.973794405 0.945292298 0.942785831 0.944601987 0.934463481

1.062301384 1.051137019 1.064545285 1.045371374 1.026120517 1.038546884 1.04490555 0.97557146 0.932192957 0.95692081 0.928445216 0.873941544

1.042373082 1.091270549 1.045853408 1.028616884 1.02259186 0.993923753 1.002378438 0.969600728 0.941316009 0.948761191 0.938547839 0.974766258

0.980850086 0.967399729 0.965980451 0.973740743 0.969975744 0.992052852 0.983600693 0.995667854 1.051320234 1.066117126 1.051930243 1.001364245

1.02512473 1.005229041 1.029859581 1.015523091 1.021006891 1.009957722 0.992340141 0.99904437 0.985263708 0.978788138 0.986907433 0.950955155

1.028136089 1.022310941 1.010702984 1.020535371 1.016872741 0.969029889 0.967979168 0.971790007 0.999547101 0.999351754 0.999673174 0.994070781

1.04403582 1.031540754 1.034477781 0.979665142 1.054538604 1.021922353 0.995954239 0.99727998 0.968265704 1.001618419 0.96886113 0.901840074

0.982347087 0.976204154 1.002278076 0.98867242 1.003071051 1.033109737 1.025610116 1.034629079 0.981930714 0.993719943 0.98046881 0.997958812];

% Labels for amino acids and species

amino_acids = {'A', 'C', 'D', 'E', 'F', 'G', 'H', 'I', 'K', 'L', ...

'M', 'N', 'P', 'Q', 'R', 'S', 'T', 'V', 'W', 'Y'};

species = {'Ca. Brocadia carolinensis','Ca. Brocadia fulgida','Ca. Brocadia pituitae','Ca. Brocadia sapporoensis','Ca. Brocadia sinica','Ca. Jettenia caeni','Ca. Jettenia ecosi','Ca. Kuenenia stuttgartiensis','Ca. Scalindua brodae','Ca. Scalindua japonica','Ca. Scalindua rubra','Ca. Scalindua sediminis'};

for i = 1:1:1000

blindmap1(i,1) = (0.9290/1000)*i;

blindmap1(i,2) = (0.6940/1000)*i;

blindmap1(i,3) = 0.8-(((0.8-0.1250)/1000)*i);

end

% Normalize the data to highlight enrichments

normalized_data = amino_acid_proportions;

% Plot heatmap

figure;

heatmap(species, amino_acids, normalized_data, 'Colormap', blindmap1, ...

'ColorbarVisible', 'on','CellLabelColor','none');

title('Amino Acid Enrichments Across Species');

xlabel('Species');

ylabel('Amino Acids');

% Perform PCA on the normalized data (transpose needed for PCA)

[coeff, score, latent, tsquared, explained] = pca(amino_acid_proportions');

% Plot explained variance

figure;

bar(explained);

xlabel('Principal Component');

ylabel('Variance Explained (%)');

title('PCA Explained Variance');

% Visualize PCA scores

figure;

scatter(score(:, 1), score(:, 2), 100, 'filled');

text(score(:, 1), score(:, 2), species, 'VerticalAlignment', 'bottom', ...

'HorizontalAlignment', 'right');

xlabel('PC1');

ylabel('PC2');

title('PCA Scores Plot');

grid on;

% Highlight amino acid contributions to PCs

figure;

biplot(coeff(:, 1:2), 'VarLabels', amino_acids);

% Add labels and title

title('Amino Acid Contributions to PCA');

xlabel(['PC1 (' num2str(explained(1), '%.2f') '%)']);

ylabel(['PC2 (' num2str(explained(2), '%.2f') '%)']);

grid on;

% Sort loadings for PC1

[sortedLoadings, idx] = sort(abs(coeff(:, 1)), 'descend');

% Display top 10 genes contributing to PC1

topGenes_PC1 = amino_acids(idx(1:5));

topLoadings_PC1 = coeff(idx(1:5), 1);

disp(table(topGenes_PC1.', topLoadings_PC1));

When run in MATLAB, this code generates 2 figures related to the PCA results and displays the amino acids with the highest PCA loadings in the command window.

**References**

Adobe Inc. 2019. “Adobe Illustrator.” https://adobe.com/products/illustrator.

Ali, Muhammad, Mamoru Oshiki, Takanori Awata, Kazuo Isobe, Zenichiro Kimura, Hiroaki Yoshikawa, Daisuke Hira, et al. 2015. “Physiological Characterization of Anaerobic Ammonium Oxidizing Bacterium ‘CandidatusJettenia Caeni.’” *Environmental Microbiology* 17 (6): 2172–89. https://doi.org/10.1111/1462-2920.12674.

Awata, Takanori, Mamoru Oshiki, Tomonori Kindaichi, Noriatsu Ozaki, Akiyoshi Ohashi, and Satoshi Okabe. 2013. “Physiological Characterization of an Anaerobic Ammonium-Oxidizing Bacterium Belonging to the ‘Candidatus Scalindua’ Group.” *Applied and Environmental Microbiology* 79 (13): 4145–48. https://doi.org/10.1128/AEM.00056-13.

Bielski, Roman G., and M. Ahsanul Islam. 2024. “Pangenome-Scale Mathematical Modelling of ANAMMOX Bacteria Metabolism.” *SynBio* 2 (1): 70–84. https://doi.org/10.3390/synbio2010005.

Narita, Yuko, Lei Zhang, Zen ichiro Kimura, Muhammad Ali, Takao Fujii, and Satoshi Okabe. 2017. “Enrichment and Physiological Characterization of an Anaerobic Ammonium-Oxidizing Bacterium ‘Candidatus Brocadia Sapporoensis.’” *Systematic and Applied Microbiology* 40 (7): 448–57. https://doi.org/10.1016/j.syapm.2017.07.004.

Oshiki, Mamoru, Masaki Shimokawa, Naoki Fujii, Hisashi Satoh, and Satoshi Okabe. 2011. “Physiological Characteristics of the Anaerobic Ammonium-Oxidizing Bacterium ‘Candidatus Brocadia Sinica.’” *Microbiology* 157 (6): 1706–13. https://doi.org/10.1099/mic.0.048595-0.

The MathWorks Inc. 2022. “MATLAB Version: 9.13.0 (R2022b).” Natick, Massachusetts, United States: The MathWorks Inc. https://www.mathworks.com.

Van Der Star, Wouter R.L., Andreea I. Miclea, Udo G.J.M. Van Dongen, Gerard Muyzer, Cristian Picioreanu, and Mark C.M. Van Loosdrecht. 2008. “The Membrane Bioreactor: A Novel Tool to Grow Anammox Bacteria as Free Cells.” *Biotechnology and Bioengineering* 101 (2). https://doi.org/10.1002/bit.21891.

Van Niftrik, Laura, Willie J.C. Geerts, Elly G. Van Donselaar, Bruno M. Humbel, Alevtyna Yakushevska, Arie J. Verkleij, Mike S.M. Jetten, and Marc Strous. 2008. “Combined Structural and Chemical Analysis of the Anammoxosome: A Membrane-Bounded Intracytoplasmic Compartment in Anammox Bacteria.” *Journal of Structural Biology* 161 (3): 401–10. https://doi.org/10.1016/j.jsb.2007.05.005.
